# Evidence based consensus statements for digital tools to address youth mental health literacy

**DOI:** 10.1038/s41598-025-12947-y

**Published:** 2025-08-02

**Authors:** Stephana Julia Moss, Sonia Siddiqui, Cristina Zuniga Chacon, Cynthia Sriskandarajah, Maia Stelfox, Ben Gaunce, Micaela Harley, Stacie Smith, Sofia B. Ahmed, Kathryn Birnie, Donna Halperin, Scott Halperin, Christine Hampson, Jia Hu, Laura Leppan, Angie Nickle, Kristine Russell, Andrea Soo, May Solis, Henry T. Stelfox, Sharon Straus, Perri R. Tutelman, Quincy Wiele, Kirsten M. Fiest, Nicole Racine, Jeanna Parsons Leigh

**Affiliations:** 1 Department of Pediatrics, Faculty of Medicine, Dalhousie University, Halifax, NS Canada; 2 School of Health Administration, Faculty of Health, Dalhousie University, Halifax, NS Canada; 3 Royal’s Institute of Mental Health Research, Ottawa, ON Canada; 4 Young Canadians Roundtable on Health, Toronto, ON Canada; 5 Faculty of Medicine and Dentistry, University of Alberta, Edmonton, AB Canada; 6 Department of Anesthesiology, Perioperative and Pain Medicine, Cumming School of Medicine, University of Calgary, Calgary, AB Canada; 7 Rankin School of Nursing, St. Francis Xavier University, Antigonish, NS Canada; 8 The Sandbox Project, Toronto, ON Canada; 9 Department of Critical Care Medicine, Cumming School of Medicine, University of Calgary, Calgary, AB Canada; 10 Institute of Health Policy, Management and Evaluation, Faculty of Medicine, University of Toronto, Toronto, ON Canada; 11 Department of Oncology, Cumming School of Medicine, University of Calgary, Calgary, AB Canada; 12 Faculty of Nursing, University of Calgary, Calgary, AB Canada; 13 School of Psychology, University of Ottawa, Ottawa, ON Canada; 14 Canadian Center for Vaccinology, Faculty of Medicine, Dalhousie University, Halifax, NS Canada

**Keywords:** Youth, Digital tool, Mental health literacy, Delphi consensus process, Focus groups, Paediatrics, Public health

## Abstract

Mental health disorders typically emerge in early life and can be modified through prompt intervention. Mental health literacy is the multi-dimensional knowledge of mental health disorders to recognize, manage, or prevent mental health disorders. Enhancing youth mental health literacy through information communication technologies already adopted by youth may be an accessible and effective approach to address the ongoing mental health crisis. We used an interconnected, three phase process following co-design methods to determine evidence-based consensus statements to conceptualize and develop the Youth MindTrack and to inform future digital tools to support youth mental health literacy. In Phase I, a scientific team (*N* = 21; 24% youth) adhered to deliberative dialogue and priority setting methods to develop strategic priorities. Phase II consisted of a modified Delphi consensus process to determine evidence-based consensus statements (*N* = 352 (13% youth) Round 1; *N* = 87 (33% youth) Round 2). Semi-structured focus groups in Phase III were conducted to refine the consensus statements (*N* = 16, 25% youth) and design the digital mental health literacy tool (*N* = 24, 33% youth). Twenty-one consensus statements encompassing four domains were produced: (1) Understanding mental health (*N* = 4); (2) Exercising mental health (*N* = 6); (3) Engaging with digital support (*N* = 8); and (4) Evaluating digital support (*N* = 3). Content analysis of discussions identified 16 themes mapped to the domains of mental health literacy and user-interface, design considerations for digital tools on mental health literacy more broadly. The resulting design of the Youth MindTrack tool to support mental health literacy included four main interactive sections designed to be downloaded and completed by youth on a digital device. We determined 21 evidence-based consensus statements that underpinned the conceptualization and development of the Youth MindTrack: a downloadable digital tool to support youth mental health literacy. The data supports proceeding to pilot testing to assess the tool’s usability, acceptability, and perceived effectiveness prior to implementation, and provides evidence that an iterative and participatory research-based process with youth can help adapt health technology to their needs.

**Supplementary Information:**

The online version contains supplementary material available at 10.1038/s41598-025-12947-y.

## Introduction

There is substantial evidence that mental health disorders frequently emerge early in life and typically demonstrate a course characterized by chronicity and numerous periods of relapse^1,2^. This course can be modified through early intervention as youth (defined as young people aged 11–18 for the purposes of this study^3^ often reveal a need for mental health care prior to reaching the threshold for a traditional psychiatric diagnosis^4–6^. Early and effective management of mental health disorders during this critical developmental period thus may help to reduce extensive morbidities and adverse outcomes, such as social isolation, decreased vocational and educational productivity, and poor functioning^7–9^. Nonetheless, youth are significantly less likely than other age group to access mental health support services due to various factors including comparatively lower mental health literacy, stigma among family or friends, inaccessible health system structures, and inadequate or inappropriate resources^10–13^. While mental ill health accounts for 45% of the disease burden among individuals aged 10 to 24, only 2% of global health budgets are allocated to mental healthcare for this age group. This disparity highlights a critical gap in addressing the escalating mental health needs of youth worldwide^14^.

Recent years have witnessed transformative advances in the sophistication and accessibility of information communication technologies for the delivery of high-quality mental health care to youth through telemedicine (i.e., practitioner-driven distribution of medical services via electronic information and telecommunication technologies)^15–17^. Broadened internet availability and increased capacities for mobile broadcasting have enhanced youth access to mental health care, effectively addressing the wide range of needs^18–20^. Enhanced service efficiency through telemedicine modalities has also reduced care gaps across low-, medium-, and high-resource settings^21–24^. Among youth, various studies have suggested that mental health care delivered through telemedicine is effective in reducing symptoms of anxiety, depression, emotional and behavioral disorders, and substance use disorders^25–29^.

However, youth must first obtain adequate knowledge of how and when mental health disorders develop to appraise their need for help and to engage in help-seeking behaviours^30,31^. Several recent systematic reviews show that perceived lack of knowledge about mental health challenges and disorders is one of the most pervasive barriers to help-seeking behaviours among youth^32–34^. Enhancing mental health literacy is one promising approach to promote help-seeking behaviours to combat excessively high rates of undertreatment of mental disorders among youth^35,36^. Mental health literacy is conceptualized as multi-dimensional knowledge of mental health disorders to recognize, manage, or prevent mental health disorders^37,38^. The several, interlinked components within the three domains that comprise mental health literacy include: Domain 1 Recognition: (1) the ability to recognize specific disorders or different types of psychological distress; Domain 2 Knowledge: (2) knowledge and beliefs about risk factors and causes, (3) knowledge and beliefs about self-help interventions, (4) knowledge and beliefs about professional help available, (5) knowledge of how to seek mental health information; and Domain 3 Attitudes: (6) attitudes that facilitate recognition and appropriate help-seeking behaviour^39–42^.

Digital tools have become increasingly instrumental to enhance mental health literacy among youth, offering scalable and engaging platforms to disseminate knowledge, reduce stigma, and promote help-seeking behaviors. A meta-analysis encompassing 29 studies with over 11,000 participants demonstrated that internet-based interventions significantly improved mental health knowledge, reduced stigma, and increased help-seeking intentions, though sustaining these effects over time remains a challenge^43,44^. Digital video interventions, such as documentaries and informational videos, have also shown promise in improving mental health literacy among young people, with studies indicating positive outcomes in at least one component of mental health literacy^44^. Innovative approaches like the IMPeTUs intervention in Indonesia have co-developed immersive digital applications with adolescents to address depression and anxiety, incorporating interactive games and exercises to promote engagement and self-management strategies^45^. Similarly, programs like MediaSmarts in Canada provide digital media literacy resources aligned with provincial curricula, aiming to equip youth with critical thinking skills to navigate digital media and its impact on mental health^46^. These initiatives underscore the potential of digital tools in fostering mental health literacy among youth, emphasizing the importance of culturally tailored, interactive, and accessible interventions.

As one step toward addressing the mental health crisis in Canada^47–50^, we aimed to co-design a digital tool to improve mental health literacy among youth aged 11–18. We also aimed to produce evidence-based consensus statements to aid the conceptualization, development, and implementation of future digital tools by other research groups to support youth mental health literacy. Intentional engagement following established co-design guidelines of diverse youth, parents, health professionals, researchers and academics, and policy- and decision-makers, throughout this multi-phased, interconnected project was crucial to our success^51^. Extensive bi-directional dialogue with core national partner organizations (i.e., Children’s Healthcare Canada, The Sandbox Project, and Young Canadians Roundtable on Health) allowed us to directly infuse youth voices within each phase of the research process as well as within core deliverables. Herein, we describe the methodology for this work, alongside presentation of the evidence-based consensus statements that underpinned the development of our co-designed Youth MindTrack: an interactive digital tool to enhance youth mental health literacy.

## Methods

**Fig. 1:**
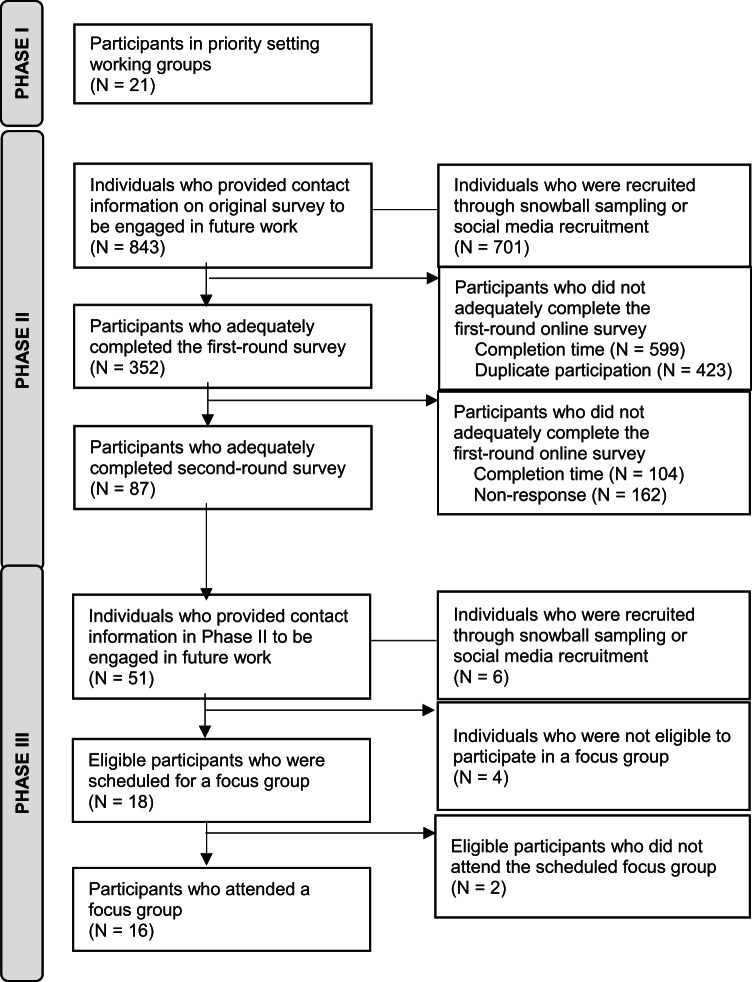
Study flow diagram.

From September 2023 to June 2024, we led an interconnected three-phase process with diverse key informants to develop and iteratively refine the Youth MindTrack, a downloadable and interactive tool to support and enhance youth mental health literacy on their digital devices. We also determined evidence-based consensus statements to aid the conceptualization, development, and implementation of future digital tools to support youth mental health literacy (Fig. 1). Following established guidelines for a six-step co-design process (i.e., engage, plan, explore, develop, decide and change)^51–53^, we partnered with youth and parents at the conception of this project; youth and family research partners were actively engaged throughout all phases of work and compensated appropriately^54^. A priori recruitment benchmarks for each phase were determined in consultation with our partner organizations (i.e., Children’s Healthcare Canada, The Sandbox Project, and Young Canadians Roundtable on Health). The ethics review boards at the University of Calgary (#23–0039) and Dalhousie University (#2023–6538) approved this project. The protocol for this work has been published previously^55^.

### Phase I: strategic priority setting working groups

To develop the strategic priorities for this overarching program of work, we convened a scientific team with expertise in child and youth developmental and socioemotional health, clinical psychology and social work, philosophy, sociology, epidemiology, applied health services research, implementation science, as well as diverse youth and parent research partners. We followed published guidance on principles for successful interdisciplinary research teams^56^. Our a priori recruitment benchmarks for Phase I included: over 20% of scientific team members aged 11–17 years (i.e., children and youth), over 20% of scientific team members self-identifying their primary role as a healthcare provider, at least 10% of scientific team members self-identifying their ethnicity as Black, Indigenous, or a Visible Minority, at least 10% of scientific team members self-identifying as an immigrant to Canada within the last five years, and at least 10% of scientific team members self-identifying with a disability (either visible or non-visible)^57–60^. We also intentionally created a broad team spanning several Canadian provinces and including multiple members external to the core research team. Over three months, following established guidelines for deliberative dialogue^61^, the scientific team interacted in person, by telephone, videoconferencing, and electronic communication within structured working groups (i.e., no primary data collection; 90 min each, three working groups in total, one working group per month) to develop the guiding strategic priorities for this project. Working groups were facilitated by the study lead (SJM) and supported by the research team (SS, MS, CS, CZC). We used the conceptual framework for priority setting proposed by Sibbald and colleagues to specify quantitative and qualitative dimensions of our priority setting process as they related to both process and outcome components^62^. The ten interconnected elements included within this framework were: (1) key informant engagement; (2) key informant understanding; (3) key informant acceptance and satisfaction; (4) consideration of values and context; (5) use of explicit process; (6) positive externalities; (7) decision making quality; (8) information management; (9) shifted priorities/reallocation of resources; and (10) and revision or appeals mechanism. Working groups were recorded (audio and video), and additional field notes were taken to reference context. Participants were provided with detailed meeting minutes and a summary of discussions after the conclusion of each working group. Following the third working group, the drafted strategic priorities were reviewed and iteratively refined by the scientific team via “live” documents housed online until no further revisions were made, at which point the strategic priorities were considered final. Youth and parent research partners who participated as members of the scientific team were provided with a $100 gift card as a thank you for their time.

### Phase II: modified Delphi consensus process

To determine evidence-based consensus statements to aid the conceptualization, development, and implementation of current and future digital tools for supporting and enhancing youth mental health literacy, we conducted a modified Delphi consensus process^63^ according to the RAND-UCLA appropriateness method^64^ and following expert recommendations for using the Delphi method in mental health research^65^, and the Conducting and REporting DElphi Studies reporting guidelines (Supplemental Table 1)^66^. We initiated recruitment by inviting youth and parents (*N* = 843, total) who provided contact information in our previous studies to be engaged in future research^67,68^. We used a convenience sample and snowballing process to recruit youth and parents to participate in the modified Delphi consensus process. Additional avenues for recruitment (i.e., advertisements in the bi-weekly Children’s Healthcare Canada newsletter or on social media platforms) were applied to obtain the experiences of subpopulations that were under-represented (i.e., according to youth gender identity or sexual orientation (LGBTQ+), youth or parent ethnicity, and parental household income) in the participant list generated from our previous studies^67,68^. Our a priori recruitment benchmarks for Phase II included: over 75% of respondents aged 11–17 years (i.e., children or youth) or aged 19 + years and self-identifying their primary role as a parent (i.e., not a researcher, decision maker, or healthcare provider), over 50% of respondents self-identifying their gender as men or boys and at least 5% of respondents self-identifying their gender as another gender identity (other than men or boys, women or girls), over 40% of respondents self-identifying their ethnicity as Black, Indigenous, or a Person of Colour, over 15% of respondents self-identifying as an immigrant to Canada within the last 5 years, at least 10% of respondents self-identifying with a disability (either visible or non-visible), and at least 5% of respondents from every province in Canada (with Atlantic provinces amalgamated into a single category)^57–60^.

Sixty-eight items were created based on our foundational evidence that included: a scoping review of strategies to mitigate the impact of public health crises on youth mental health^69^, a scoping review of theories, models, and frameworks that have been used to implement digital tools targeted to youth mental health^70^, a systematic review of mental health interventions to support youth recovery from the COVID-19 pandemic^71^, a national cross-sectional survey of social factors associated with self-reported changes in mental health symptoms among youth during the COVID-19 pandemic^67^, a national cross-sectional survey on mental health literacy including information sources accessed or preferred among parent-youth dyads during the COVID-19 pandemic^68^, and semi-structured interviews with parent-youth dyads on their experiences with mental health symptoms during the COVID-19 pandemic^72^. Bibliographies of key papers on digital tools to support youth mental health^73–77^ and broader interventions and initiatives to enhance youth mental health literacy^78–83^ were reviewed independently and in-duplicate by two study authors (SJM, CZC) to locate additional relevant data for inclusion. The 68 items were organized into three domains and eight themes: Domain 1 Psychosocial Education on Youth Mental Health: (1) influential factors for youth mental health (*n* = 11 items), (2) identification of symptoms for youth mental health (*n* = 10 items); Domain 2 Conduction of Youth Mental Health Care: (3) preventive interventions to mitigate youth mental health disorders (*n* = 13 items), (4) reactive interventions to address youth mental health disorders (*n* = 5 items), (5) self-guided strategies and approaches for youth mental health disorders (*n* = 5 items); and Domain 3 Dissemination of a Digital Tool for Youth Mental Health (6) key informants to engage in digital youth mental health tool research (*n* = 6 items), (7) strategies for implementation of digital youth mental health tools (*n* = 14 items), and (8) evaluation of success for digital youth mental health tools (*n* = 4 items).

Participants assessed and rated individual items using a validated 9-point Likert scale (1 = non-essential, 9 = essential) to evaluate their importance in relation to digital tools for supporting and enhancing mental health literacy among youth^63,84,85^. This involved two online survey rounds of modified Delphi consensus voting. Both rounds were self-administered, using a secure and encrypted online platform (Qualtrics, Provo, UT). The overall modified Delphi process is shown in Supplemental Fig. 1. All surveys were developed and pilot-tested by team members, youth and parent partners, policy- and decision-makers, and healthcare providers to ensure that the total time to completion did not exceed 20 min, and that questions were age and grade-level appropriate, clear, and comprehensive. Eligible participants provided informed consent prior to participating in each round of the modified Delphi consensus process. Given that the total time to completion for both online surveys was at minimum 15 min, survey submissions marked as completed in under five minutes were excluded to ensure veracity of the data^86^.

We determined consensus for any statement a priori as a median score of 1–3 (not essential) or 7–9 (essential). Two weeks after the first round, we emailed participants a summary of results that included the median score and interquartile range for each item, a list of the items that reached consensus as being not essential or essential (and thus were excluded from the second round), and additional items as suggested by participants; this email marked the commencement of the second online survey round that remained open for two full weeks. We emailed participants the same summary after the second round except for additional items as suggested by participants (given the option to do so was not provided in the second online survey). Individual items and associated scores for each round are provided in Appendix 1. Analyses were conducted using Microsoft Excel (V16.83). Participants who completed both rounds of the modified Delphi consensus process were given a $50 gift card as a thank you for their time. In lieu of a third online survey round, we conducted serial focus groups (described below, Phase III).

### Phase III: consensus statement refinement and tool design focus groups and partnership meetings

Serial focus groups were conducted by the study lead (SJM) or a sub-set of the research team (SS, CZC), to review, revise, and refine the consensus statements from Phase II (focus groups #1-#3), and to provide feedback on consecutive prototypes of the digital tool (focus groups #4-#7) (specific focus group objectives provided in Supplemental Table 2). We did not apply recruitment benchmarks in Phase III; instead, we adjusted our study protocol to ensure all those interested in participating in a focus group were given the opportunity to do so. Development of the digital tool began in focus group #4, with revisions to the tool occurring after each subsequent focus group. Three groups of key informants were recruited to participate in seven focus groups that included: (1) youth and parents (three focus groups; #1, #4, #7), (2) academics and researchers (two focus groups; #2, #5); and (3) healthcare providers (two focus groups; #3, #6)^87^. Discussions in focus groups #4-#7 were grounded in four key considerations: (1) the tool must be useful from the youth’s perspective; (2) the tool must be easy to learn and master for youth between the ages of 11 and 18 years; (3) the tool must be applicable to a broad range of mental health symptoms that are associated with various mental health disorders, rather than narrowly focused on one (or few) specific mental health disorders; and 4) the tool must be inexpensive, able to be individualized, and scalable to diverse youth broadly. We followed established guidelines for participatory research processes with youth as well as recommendations from other groups on co-designing interventions with youth to ensure youth felt that they were heard, seen, and offered various opportunities to actively participate in meaningful and accessible ways^88–92^. Recruitment was initiated by emailing all participants who completed both rounds of the Delphi consensus process (*N* = 87); purposive sampling was employed to increase diversity in youth age, sex, and gender. Youth were not required to participate with a parent, and vice versa; we did not exclude them if they did. All focus group participants received a $25 gift card as a thank you for their time.

To further infuse youth voices and perspectives throughout Phase III, we partnered with the Young Canadian Roundtable on Health (a national youth engagement and education organization working to connect youth with policymakers to address gaps in youth health), and The Sandbox Project (that aims to help organizations come together, share resources, and leverage partnerships to address specific health challenges faced by youth in Canada). We held three partnership-based meetings involving a total of nine young participants (three youth per meeting) from the Young Canadian Roundtable on Health and two expert partners (both present at all meetings) from The Sandbox Project: the first meeting (led by SJM) was conducted after the first set of three focus groups to interpret feedback on the consensus statements obtained from focus groups #1-#3; the second meeting (led by SS) was conducted after the second set of three focus groups (#4-#6) to aid our interpretation of the collected qualitative data on feedback for the digital tool prototype; and the third meeting (led by SS) was conducted after the last focus group (#7) to aid our interpretation of the collected qualitative data on feedback for the final digital tool. Additionally, youth partners reviewed the facilitator guides and objectives for each focus group prior to conduction.

**Fig. 2:**
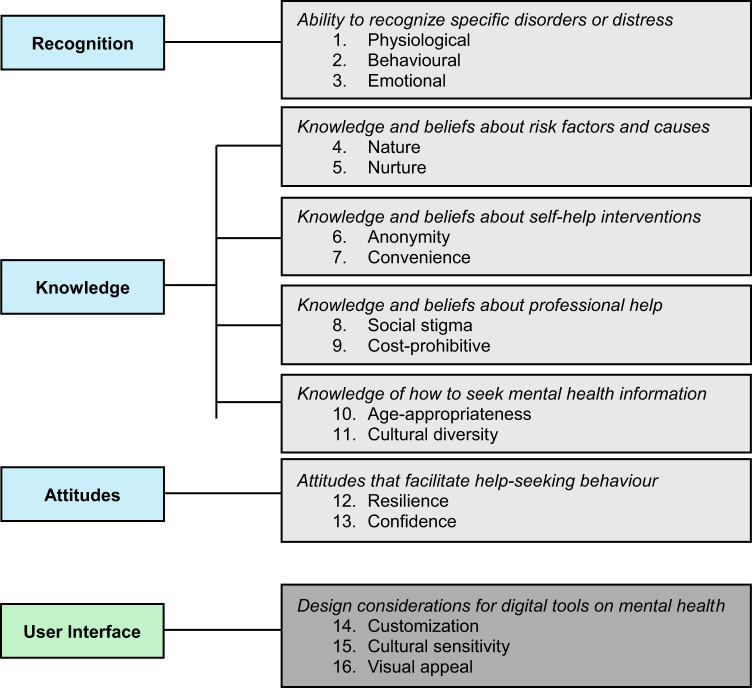
Phase III content and design themes mapped to the domains that comprise mental health literacy and user interface considerations respectively.

Focus group data were analyzed using content analysis^93^ and managed through NVivo 12 software (QSR International, Melbourne Australia). We employed an inductive approach to gain information directly from the data without imposing preconceived theoretical perspectives^94^. Focus group transcripts were independently read in duplicate by two authors (SJM, SS) to grasp the content and identify units of meaning corresponding to the aims of Phase III; content of the transcripts was coded according to meaning. The authors (SJM, SS) kept an audit trail to track the evolving analysis and used a reflexive journal to enhance the study’s methodological rigor^95^. The resulting codes were then organized into thematic categories and mapped to the domains that comprise mental health literacy (described above): Domain 1 Recognition; Domain 2 Knowledge; and Domain 3 Attitudes (Fig. 2)^39–42^.

## Results

### Phase I: strategic priority setting working groups

#### Sample characteristics

We convened a scientific team that included children (11–14 years of age; *n* = 2, 9.5%), youth (15–18 years of age; *n* = 3, 14.3%), and a parent (19 + years of age; *n* = 1, 4.8%), as well as researchers (*n* = 8, 38.1%), decision makers (*n* = 2, 9.5%), and healthcare providers (*n* = 5, 23.8%); the majority of the team self-identified as women(*n* = 16, 76.2%) and White (*n* = 18, 85.7%). A third of the team was from Alberta (*n* = 7, 33.3%), a third from the Atlantic provinces (*n* = 7, 33.3%), with the remaining members of the team from Ontario (*n* = 8, 38.1%). Table 1 presents participant demographics and characteristics by phase of study.

**Table 1:** Participant demographics and characteristics by phase of study.

Demographics	Phase I Strategic priorities (*N* = 21)	Phase II DELPHI Consensus		Phase III Focus groups (*N* = 16)
		Round 1 (*N* = 352)	Round 2 (*N* = 87)	
Participant type (N, %)				
Child (11–14 years)	2 (9.5%)	6 (1.7%)	6 (6.9%)	2 (12.5%)
Youth (15–18 years)	3 (14.3%)	43 (12.2%)	23 (26.4%)	2 (12.5%)
Parent (19 + years)	1 (4.8%)	268 (76.1%)	56 (64.4%)	4 (25.0%)
Researcher	8 (38.1%)	19 (5.4%)	1 (1.2%)	5 (31.3%)
Decision maker	2 (9.5%)	9 (2.6%)	0 (0.0%)	0 (0.0%)
Healthcare provider	5 (23.8%)	16 (4.6%)	1 (1.2%)	3 (18.8%)
Age category (N, %)				
11–14 years	2 (9.5%)	6 (1.7%)	6 (6.9%)	2 (12.5%)
15–18 years	3 (14.3%)	43 (12.2%)	23 (26.4%)	2 (12.5%)
19–30 years	6 (28.6%)	60 (17.1%)	14 (16.1%)	8 (50.0%)
31–50 years	6 (28.6%)	215 (61.1%)	31 (35.6%)	4 (25.0%)
51–64 years	4 (19.0%)	26 (7.4%)	11 (12.6%)	0 (0.0%)
65 + years	0 (0.0%)	2 (0.6%)	2 (2.3%)	0 (0.0%)
Gender (N, %)				
Woman or girl	16 (76.2%)	143 (40.6%)	38 (43.7%)	8 (50.0%)
Man or boy	5 (23.8%)	192 (54.6%)	45 (51.7%)	8 (50.0%)
Another gender identity	0 (0.0%)	17 (4.8%)	4 (4.6%)	0 (0.0%)
Province (N, %)				
British Columbia	0 (0.0%)	54 (15.3%)	17 (19.5%)	2 (12.5%)
Alberta	7 (33.3%)	32(9.1%)	12 (13.8%)	4 (25.0%)
Saskatchewan or Manitoba	0 (0.0%)	25 (7.1%)	6 (6.9%)	0 (0.0%)
Ontario	8 (38.1%)	109 (30.9%)	36 (41.4%)	5 (31.3%)
Quebec	0 (0.0%)	82 (23.3%)	8 (9.2%)	0 (0.0%)
Atlantic provinces	7 (33.3%)	50 (14.2%)	8 (9.2%)	5 (31.3%)
Territories	0 (0.0%)	0 (0.0%)	0 (0.0%)	0 (0.0%)
Ethnicity (N, %)				
White	18 (85.7%)	200 (56.8%)	58 (66.7%)	8 (50.0%)
Black, Indigenous, or Person of Colour	3 (14.3%)	152 (43.2%)	29 (33.3%)	6 (37.5%)
Other	0 (0.0%)	0 (0.0%)	0 (0.0%)	2 (12.5%)
Education (N, %)				
High school	1 (4.8%)	45 (12.8%)	14 (16.1%)	1 (6.3%)
College diploma	0 (0.0%)	89 (25.3%)	7 (8.0%)	1 (6.3%)
Undergraduate	3 (14.3%)	102 (29.0%)	29 (33.3%)	2 (12.5%)
Graduate	15 (71.4%)	67 (19.0%)	8 (9.2%)	8 (50.0%)
Immigrant (N, %)^a^				
Yes	2 (9.5%)	74 (21.0%)	14 (16.1%)	2 (12.5%)
Disability (N, %)				
Yes, visible	1 (4.8%)	24 (6.8%)	2 (2.3%)	0 (0.0%)
Yes, non-visible	3 (14.3%)	51 (14.5%)	5 (5.8%)	1 (6.3%)

N, number; N/A, not applicable.

^a^Within the last five years.

#### Strategic priorities

**Fig. 3:**
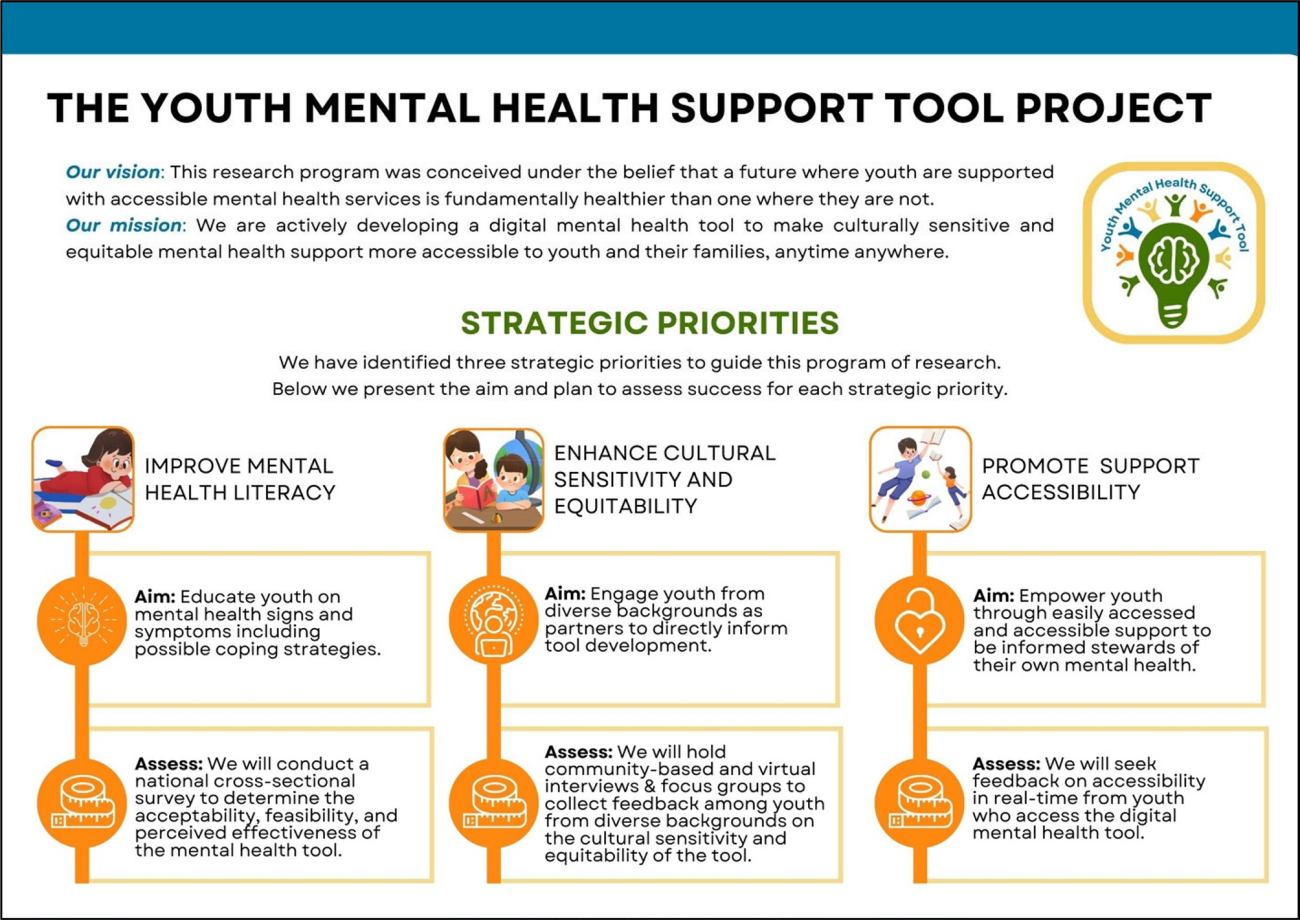
Phase I strategic priorities.

The vision, mission, and strategic priorities that were developed through our working groups are illustrated in Fig. 3. The scientific team identified three strategic priorities to guide this program of research: (1) Improve mental health literacy (aim: educate youth on mental health signs and symptoms, including possible coping strategies); (2) Enhance cultural sensitivity and equitability (aim: engage youth from diverse backgrounds as partners to directly inform tool development); (3) Promote support accessibility (aim: empower youth through easily accessed and accessible support to be informed stewards of their own mental health). The scientific team also determined three approaches to assess success for each strategic priority that consisted of a cross-sectional survey to pilot test the digital tool (Priority 1), collection of qualitative data on tool cultural sensitivity and equity (Priority 2), and online, short-form feedback from youth who access the digital tool in real-time (Priority 3).

### Phase II: modified Delphi consensus process

#### Sample characteristics

We received 1,374 completed submissions to the first round of the modified Delphi consensus process from individuals who engaged with our previous work on this topic (*N* = 673/843, 79.8%) or from snowball sampling or social media recruitment (*N* = 701, 100.0%). Of these, 352 (22.8%) were considered valid submissions, meaning that they were not duplicate submissions (*N* = 423, 31.9% excluded) or submissions that were completed in under five minutes (*N* = 599, 45.3% excluded). After contacting the 352 participants associated with valid submissions in Round 1, we received 87 valid submissions in Round 2 once we excluded submissions that were completed in under five minutes (*N* = 104, 54.5% of completed submissions); 162 (46.1%) of participants from round one did not respond to our request to complete round two.

Participants in Round 1 were mostly parents (*N* = 269, 76.1%) or youth (*N* = 43, 12.2%), self-identified as a man or boy (*N* = 192, 54.6%), and resided in Ontario (*N* = 109, 30.9%) or Quebec (*N* = 82, 23.3%). Many participants in Round 1 self-identified as Black, Indigenous, or a Visible Minority (*N* = 152, 43.2%), and held an undergraduate degree (*N* = 102, 29.0%) or college diploma (*N* = 89, 25.3%). Nearly one quarter of participants in round one self-identified as an immigrant to Canada within the last five years (*N* = 74, 21.0%). Participants in Round 2 were mostly parents (*N* = 56, 64.4%) or youth (*N* = 23, 26.4%), self-identified as a man or boy (*N* = 45, 51.7%), and resided in Ontario (*N* = 36, 41.4%) or British Columbia (*N* = 17, 19.5%). One third of participants in Round 2 self-identified Black, Indigenous, or a Visible Minority (*N* = 29, 33.3%), and held an undergraduate degree (*N* = 29, 33.3%).

#### Consensus statement determination

Results from the modified Delphi consensus process by round, domain, and theme are provided in Supplemental Table 3. Among adults who completed Round 1, 33.3% (7/21) of statements in Domain 1 (Psychosocial Education on Youth Mental Health), 78.3% (18/23) of statements in Domain 2 (Provision of Youth Mental Health Care), and 16.7% (4/24) of statements in Doman 3 (Dissemination of a Digital Tool for Mental Health Literacy) reached consensus. No statements (0/14) in Theme 7 (Strategies for Implementation) of Domain 3 (Dissemination of a Digital Tool for Mental Health Literacy) reached consensus among adults. Several new statements were suggested by adults in Round 1 (23 across all Domains), primarily within Theme 7 (Strategies for Implementation, 7 new statements) of Domain 3 (Dissemination of a Digital Tool for Mental Health Literacy, 10 new statements total). Among youth who completed Round 1, 61.9% (13/21) of statements in Domain 1 (Psychosocial Education on Youth Mental Health), 82.6% (19/23) of statements in Domain 2 (Provision of Youth Mental Health Care), and 70.8% (17/24) of statements in Domain 3 (Dissemination of a Digital Tool for Mental Health Literacy) reached consensus. In contrast to adults, 71.4% (10/14) of statements in Theme 7 (Strategies for Implementation) of Domain 3 (Dissemination of a Digital Tool for Mental Health Literacy) reached consensus among youth. Many statements were suggested by youth in Round 1 (22 across all domains), primarily within Theme 1 (Influential Factors for Youth Mental Health, 9 new statements) of Domain 1 (Psychosocial Education on Youth Mental Health, 13 new statements total).

With the 39 statements that did not reach consensus, and the 23 new statements as suggested by adults in Round 1, adults rated 61 statements in Round 2, ultimately reaching consensus on 42 (68.9%) statements and excluding 19 (31.1%) statements. Adults mostly excluded statements in Theme 1 (Influential Factors for Youth Mental Health; 8 statements excluded, 57.1%) of Domain 1 (Psychosocial Education on Youth Mental Health; 11 statements excluded total, 55.0%), and Theme 3 (Preventative Interventions to Mitigate Youth Mental Health Disorders; 4 excluded statements (57.1%) of Domain 2 (Provision of Youth Mental Health Care; 5 excluded statements total, 45.5%). Youth rated 45 statements in Round 2, which included 19 statements that did not reach consensus, and 26 new statements as suggested in Round 1. Youth reached consensus on 37 (82.2%) statements and excluded 8 (17.8%) statements; excluded statements were mostly in Theme 1 (Influential Factors for Youth Mental Health; 4 excluded statements, 28.9%) of Domain 1 (Psychosocial Education on Youth Mental Health; 5 excluded statements total, 23.8%), and Theme 3 (Preventative Interventions to Mitigate Youth Mental Health Disorders; 2 excluded statements, 50.0%) of Domain 2 (Provision of Youth Mental Health Care; 2 excluded statements total, 18.2%).

### Phase III: consensus statement refinement and tool design focus groups and partnership meetings

#### Sample characteristics

From the 51 participants in Phase II who provided contact information to be engaged in future work, and the six individuals who were recruited through snowball sampling or social media recruitment, we included 16 participants in serial focus groups that included two children (11–14 years, 12.5%), two youth (15–18 years, 12.5%), four parents (25.0%), five researchers (31.3%), and three healthcare providers (18.8%). Eight participants self-identified as a woman or a girl (*N* = 8, 50.0%) and eight participants self-identified as a man or a boy (*N* = 8. 50.0%). Participants were mostly White (*N* = 8, 50.0%), resided in Ontario (*N* = 5, 31.3%) or the Atlantic provinces (*N* = 5, 31.3%), and half of the sample held a graduate degree (*N* = 8, 50.0%).

#### Consensus statement refinement

We conducted three focus groups and one partnership meeting with the overarching objective to review, revise, and refine the consensus statements determined in Phase II (Supplemental Table 2). Recommendations from participants and suggested revisions to the consensus statements were presented in each subsequent meeting to iteratively refine the consensus statements according to the feedback we received. Overall, participants identified that the individual consensus statements and the Domains in which they were contained could be improved by focusing on four primary areas as related first to the statements: (1) Cohesion (i.e., consolidating similar statements as appropriate to enhance overall cohesion), (2) Accessibility (i.e., addressing accessibility gaps by amending statements to be more inclusive), (3) Uniformity (i.e., amalgamating youth and parent statements while ensuring broad age-appropriateness), and second to the Domains: (4) Simplicity (i.e., simplifying Domain names to promote ease of understanding and straightforwardness). The final, 21 consensus statements and four Domains are provided in Table 2.

**Table 2:** Phase II consensus statements by domain.

Domain	Statement
Understanding mental health	Youth mental health is a component of their overall health and wellness that interacts and is influenced by other components of their health and wellness
	Youth mental health can be influenced by an array of individual, community, and systemic factors, both in-person and online
	Symptoms of mental health struggles or disorders in youth can evolve over time as youth expand their knowledge on mental health and process their mental health experiences
	Mental health struggles or disorders among youth can manifest in physical, emotional, relational, or occupational symptoms
Exercising mental health	Mental health education for youth should include scientific, evidence-based information on coping strategies in addition to information on various treatment’s effectiveness and adherence
	Youth should be empowered to actively participate in routine assessment of their own mental health including reflection on the mental health strategies that they have adopted and implemented
	Protective social factors such as community cohesion can promote positive adaptation to mental health stressors and overall functioning among youth, particularly in situations of heightened adversity
	Early engagement in practices to support mental health in the family unit can positively impact long-term youth mental health
	Mental health practices among youth encompasses embracing balanced lifestyles, with a focus on nurturing healthy habits like regulated sleep patterns, nutritious diets, and adequate physical activity
	Promoting discussion about and self-reflection on mental health among youth is essential to transform mental health conversation into a regular and normalized practice
Engaging with mental health support	Family physicians at regular appointments can be a first touch-point with youth regarding their mental health to meaningfully contribute to promoting positive mental health practices and engaging additional support resources as necessary
	Mental health support for youth should integrate components that are adaptable to youth preferences and needs
	The development of mental health support for youth should be interdisciplinary, including healthcare providers, educators, researchers, and decision-makers, as well as youth and their families
	Mental health support for youth should be easily accessible, cost-effective, inclusive, and culturally-sensitive
	Mental health support for youth should be age-appropriate and relevant to promote continuous uptake and empowerment
	Supporting youth mental health should encompass approaches that strengthen family bonds and encourage social connections among the family
	Mental health support for youth that is online should include features for safe peer connection such as messaging boards or direct messaging functionalities
	Mental health support for youth that is online should be provided with clear guidelines and information on formal resources to access for mental health intervention
Evaluating digital mental health support	A digital mental health tool should enable youth to set and track personal goals while ensuring privacy and confidentiality
	A digital mental health tool is not meant to replicate or replace in-person mental health service methods, rather seen as an extension of in-person treatment
	A digital mental health tool should be routinely evaluated to assess fidelity, consistency, accuracy, and impact among youth

#### Tool development

The remaining four focus groups and two partnership meetings focused on varied objectives (Supplemental Table 2), all with the end goal of developing a single digital tool to support and enhance youth mental health literacy. Content analysis of discussions identified 16 themes that were subsequently mapped to the domains that comprise mental health literacy, which included, Domain 1 Recognition: (1) the ability to recognize specific disorders or different types of psychological distress [physiological; behavioural; emotional], Domain 2 Knowledge: (2) knowledge and beliefs about risk factors and causes [nature; nurture], (3) knowledge and beliefs about self-help interventions [anonymity; convenience], (4) knowledge and beliefs about professional help available [social stigma; cost-prohibitive], (5) knowledge of how to seek mental health information [age-appropriateness, cultural diversity], and Domain 3 Attitudes: (6) attitudes that facilitate recognition and appropriate help-seeking behaviour [resilience; confidence], as well as user interface, design considerations for digital tools on mental health literacy [customization, cultural sensitivity, visual appeal] (Fig. 2).

**Fig. 4:**
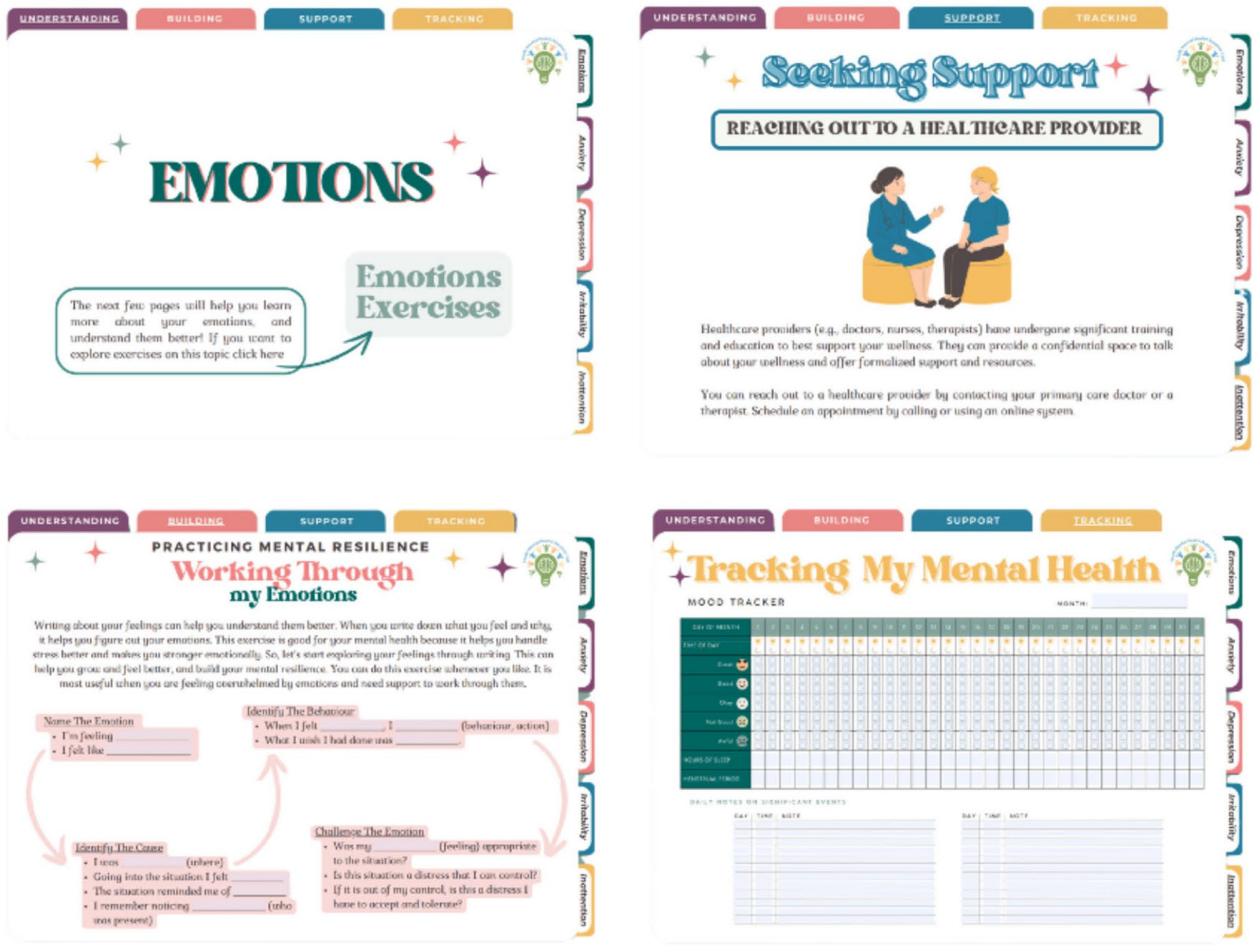
Phase III digital tool overview.

The resulting design of the Youth MindTrack (summary illustrated in Fig. 4) included four main sections designed to be downloaded and completed by youth on a digital device (e.g., tablet, computer, cell phone). These sections were driven by our strategic priorities established in Phase I, and conceptually grounded in the domains that comprise mental health literacy^39–42^, the consensus statements determined in Phase II, qualitative data collected from participants in Phase III, and our extensive foundational work in this area^67,68,96,97^. The first section was on “*Understanding* My Mental Health” that was divided into five sub-sections: (1) Emotions, (2) Anxiety, (3) Depression, (4) Irritability, and (5) Inattention; these sub-sections were selected based on ours and others earlier work to determine prevalence, relevance, and feasibility to address digitally^25–29,67,68^. Sub-section 1 presented a broad overview of how and why we feel emotions, while sub-sections 2–5 provided clinical and lay term definitions, symptoms (that are experienced in the body and the mind), and suggestions on when one might want to reach out for additional support. “*Building* My Mental Strength” was the second section that included evidence-based exercises (e.g., Grounding Method, Box Breathing, Pomodoro Technique) for users to employ in real-time^98–100^; this section was divided into the same five sub-sections (listed above) with each sub-section having two specific exercises. In the third section, we covered “Seeking *Support*,” which included information on the benefits of reaching out to a family, friend, or trusted adult, or reaching out to a healthcare provider, as well as accessible and simple steps on how to start the conversation when seeking support. This section also included a “Nationwide Resources” page where the user could access an up-to-date list of national and provincial mental health resources by selecting their home province on a map of Canada; resources included helplines, community-based services, specialized or referral-based services, and other wellness supports. The fourth section focused on “*Tracking* My Mental Health” using our “Habit Tracker” and “Mood Tracker” to increase self-awareness, identify potential triggers, and to monitor their progress. These trackers were developed with the intention for users to take proactive steps to managing their mental health and/or to facilitate ease of communication when starting a conversation to seek support. While the tool was designed to be downloaded and accessed on a digital device, it was not a static interface. The Youth MindTrack incorporated several interactive features designed specifically to engage youth users in meaningful and sustained ways. These included: personalization options (to adapt the design aesthetic of the tool to match youth preferences), gamified tracking elements (to increase motivation and sustained interaction), and opportunities for user reflection and input (such as embedded journaling prompts).

Navigation within the tool consists of a top bar and side bar menu, and a simple “home” button. The top bar menu lists all sections in the tool to promote free navigation by the user. The side bar menu lists the sub-sections contained within the “*Understanding*” section (i.e., Emotions, Anxiety, Depression, Irritability, Inattention); the landing page for each sub-section contains a link to relevant exercises within the “Building” section. The logo for this study serves as the “home” button to allow users to return to the home page at any point throughout the tool. We also intentionally designed the tool to have “fillable” fields to engage users completing exercises within the “*Building*” section, and to allow users the ability to complete the “Habit Tracker” and “Mood Tracker” online. Users can also select from various colour schemes: colourful, natural, and back-and-white.

## Discussion

Using extensive engagement that followed established co-design guidelines for diverse youth, parents, health professionals, researchers and academics, and policy- and decision-makers, we successfully developed a scalable and interactive digital tool to support and enhance mental health literacy among youth. We also produced evidence-based consensus statements to aid the conceptualization, development, and implementation of current and future digital tools for youth mental health literacy. Youth voices were directly included in all phases of this interconnected program of work and enriched through ongoing dialogue with core partner organizations. We are actively conducting a national pilot test study to assess the usability, acceptability, and perceived effectiveness of the Youth MindTrack using cross-sectional survey- and semi-structured interview-based methodologies. Overall, our rigorous methodological approaches, driven by established and relevant guidelines and frameworks, provide robust evidence that an iterative and participatory research-based process with youth can help adapt health technology to their unique mental health needs. We have contributed to the evolving field of digital mental health literacy by demonstrating how digital mental health literacy can be operationalized in a user-centered and developmentally appropriate way for youth. By embedding key emotional and cognitive domains (e.g., depression, anxiety, inattention) into an app-based format, and by aligning content with youth preferences and usage patterns, our work highlights the importance of integrating digital engagement strategies into mental health literacy efforts. Our study also illustrates how co-design, and iterative development can enhance both usability and the relevance of digital mental health interventions to advance the conceptualization and practical implementation of digital mental health literacy.

Compared to research on traditional forms of in-person or virtual, face-to-face mental health interventions among youth^101–106^, research on digital mental health literacy tools as interventions that leverage youth-adopted information communication technologies is nascent^75,107,108^. Leaders in this space have highlighted the need for studies to better describe the conceptualization and development processes for digital mental health literacy tools targeted to youth to ensure they are scalable, accessible cost-effective, and maximize research^76,109,110^. These facets are considered altogether as weaknesses of traditional face-to-face mental health interventions among all ages^105,111^. Previous studies that have documented the conceptualization and development of digital mental health literacy tools for youth have not always clearly delineated which of the three domains that comprise mental health literacy (i.e., recognition, knowledge, attitudes) are addressed in the resulting tool^112,113^. This shortcoming in our evidence base has limited subsequent examination of how these domains work conjointly within interventions, or how they relate to mental health more broadly – in particular, whether certain domains are essential (or not), and what combinations of domains yield stronger impacts (and how)^39,114,115^. To address these prominent knowledge gaps related to digital mental health literacy tools for youth, we underpinned the conceptualization of the Youth MindTrack in extensive foundational research, optimized its development through comprehensive engagement with key informants including diverse youth, and designed the tool to be readily scalable to maximize public health impact if the tool is proven efficacious. In addition the Youth MindTrack strategically integrates all domains of mental health literacy to facilitate future inquiry on the potentially moderating effect of both explicit and implicit mental health stigma (reported previously as correlated to the third domain of mental health literacy (i.e., attitudes))^116,117^. We are well positioned to investigate the impact of the Youth MindTrack on both mental health literacy and outcomes related to mental health disorders. This will add to the evidence indicating that the change process involves mental health literacy as a proximal outcome, which in turn affects various distal outcomes related to mental health disorders^118–120^. The results reported from the present work lend credence to co-designed and comprehensive digital mental health literacy tools as potentially scalable interventions that may translate to real-world mental health benefits in bolstering youth mental health functioning.

Existing evidence suggests concerns for the sustainability of digital mental health literacy interventions that target youth diagnosed with mental health disorders rather than acknowledging that one may experience subclinical symptoms of mental health disorders to address the continuum of mental health literacy more broadly^121–126^. The Youth MindTrack is novel in that it was designed to be applicable to youth who have been diagnosed with a clinical mental health disorder as well as youth who may be experiencing subclinical symptoms of mental health disorders. We accomplished this in three main ways. First, we included a sub-section specific to how and why we feel emotions, including evidence-based strategies for emotion regulation, which is an essential, yet conventionally underemphasized, feature of mental health. Earlier work has highlighted the role of emotion regulation in both clinical^127–129^ and subclinical^130–132^ symptoms of mental disorders in youth. Second, the section on seeking support that includes information on the benefits of, and how to seek support, was developed for youth to consider at any point within their mental health journey. The importance of creating a caring climate for youth has been underscored as paramount to fostering positive mental health^133–135^. The Nationwide Resources page in this section also incorporates a comprehensive list of helplines and community-based services, as well as specialized or referral-based clinical services, among other wellness supports. Third, we created two interactive trackers for youth to track their habits and mood to monitor personal progress, identify potential triggers, and increase self-awareness. These trackers were developed with the intention for youth to proactively manage mental health symptoms (whether clinical^136,137^ or subclinical^138,139^, and to serve as a data-driven resource for youth to access and reference when prompting a conversation on their mental health. Youth value self-reliance and control when accessing digital mental health tools^140–142^, which are often perceived as impersonal and unresponsive to their individual needs^143,144^. Ultimately, the potential use of the Youth MindTrack among all youth is arguably a major advance over disorder-specific digital mental health literacy tools, as it obviates the need to develop discrete mental health literacy tools for each clinical condition that impacts mental health.

### Limitations

There are several limitations to this study. First, over 800 youth and parents who completed one of our prior studies on this topic were contacted to be recruited into the modified Delphi consensus process (79.8% response, 673/843). It is possible that this group’s previous experiences working with the research team could have heightened their mental health literacy. To ensure that individuals with varied mental health literacy levels participated in the modified Delphi consensus process, we conducted snowball sampling and social media advertising to recruit an additional 701 participants. Despite that only 25.6% (352/1,374) of the total completed submissions in the first online survey round were considered valid, we did not anticipate that responses would have differed importantly by recruitment approach. Second, the retention of individuals from the first online survey round to the second online survey round of the modified Delphi consensus process was low (24.7% overall). We pilot tested the surveys with several individuals to identify and mitigate potential language barriers and to reduce time to completion (to a maximum of 20 min)^145^; we also ensured that the time between rounds (two weeks) was acceptable to prevent diminished interest and that a sufficient window of opportunity to complete the survey (two weeks) was provided^146^. The proportion of youth who completed valid submissions increased from the first survey round (13.2%) to the second survey round (33.3%), and youth (*n* = 6) were engaged in serial focus groups to provide feedback on and iteratively refine the consensus statements produced from the surveys. Nonetheless, it is unknown whether increased retention between the online survey rounds might have enhanced representativeness and permitted identification of more (or less) consensus statements. Third, we used research staff to orient focus group participants and attendees in partnership meetings to the digital tool. For maximum future scalability, this step should be performed by a youth research partner. Fourth, though not an objective of this study, it remains unclear how the tool will fit into the day-to-day lives of youth. According to Griner and Smith, culturally adapted interventions were four times more effective when tailored to a specific cultural group compared with mixed-race participant groups, and interventions conducted in one’s native language (if other than English) were twice as effective as interventions conducted in English^147^. Dedicated research is needed to engage diverse cultural groups of youth to ensure that the Youth MindTrack integrates components that are adaptable to youth preferences and needs in a youth-oriented strategy to support mental health literacy^148^.

More granular work is needed in future studies on the Youth MindTrack to ascertain barriers and facilitators to effective implementation. Fifth, while this study provides proof-of-concept that it is possible to co-design a digital tool to support and enhance youth mental health literacy, it remains to be determined whether the tool will be widely used and if used, improve clinical outcomes. Absence of consolidated evidence for the clinical effectiveness of digital mental health literacy interventions is considered a primary roadblock for the widespread promotion of mental health literacy interventions as a form of mental health disorder prevention on a global scale^149,150^. Our future studies using the Youth MindTrack will address this pervasive barrier.

## Conclusions

We successfully developed the Youth MindTrack using extensive key informant engagement and a user-centered co-design process that followed established guidelines for each phase of work. The Youth MindTrack is a digital tool to support youth in strengthening their mental health literacy that includes recognizing, managing, and preventing mental health disorders. The evidence-based consensus statements that underpinned the conceptualization and development of the tool will be useful to widespread research initiatives that seek to enhance youth mental health literacy. The data support proceeding to pilot testing to assess the tool’s usability, acceptability, and perceived effectiveness prior to implementation. Overall, this work provides evidence that an iterative and participatory research-based process with youth can help adapt health technology to their needs.

## Supplementary Information

Below is the link to the electronic supplementary material.

Supplementary Material 1

## Data Availability

The datasets generated and analysed are not publicly available as we did not secure direct permission from the survey respondents to share the de-identified dataset with the general public. Requests for the data can be directed to the institutional research ethics boards overseeing the conduct of the study via the corresponding author, Dr. Stephana Julia Moss.
