# Supplementary material for: Evidence based consensus statements for digital tools to address youth mental health literacy
Source: Sci Rep. 2025 Aug 2;15:28208. doi: 10.1038/s41598-025-12947-y (PMC12318097; doi:10.1038/s41598-025-12947-y)
Supplement: Supplementary file 1 — Supplementary Material 1 [file 41598_2025_12947_MOESM1_ESM.docx]

**Supplemental Table 1:** Conducting and REporting DElphi Studies (CREDES) Checklist

| **Section/Topic** | **#** | **Item Description** | **Page Reported** |
| --- | --- | --- | --- |
| ***Rationale for the choice of the Delphi technique*** | | | |
| *Justification* | 1 | The choice of the Delphi technique as a method of systematically building consensus needs to be well justified. | 4 |
| ***Planning and design*** | | | |
| *Planning and process* | 2 | The Delphi technique is a flexible method and can be adjusted to the respective research aims and purposes. Any modifications should be justified by a rationale and be applied systematically and rigorously. | 4 |
| *Definition of consensus* | 3 | Unless not reasonable due to the explorative nature of the study, an a priori criterion for consensus should be defined. This includes a clear and transparent guide for action on (a) how to proceed with items or topics in the next round, (b) threshold to terminate the Delphi process and (c) procedures to follow when consensus is (not) reached after one or more iterations. | 5 |
| ***Study conduct*** | | | |
| *Informational input* | 4 | All material provided to the expert panel at the outset of the project and throughout the Delphi process should be carefully reviewed and piloted in advance in order to examine the effect on experts’ judgements and to prevent bias. | 5 |
| *Prevention of bias* | 5 | Researchers need to take measures to avoid influencing the experts’ judgements. If one or more members of the research team have a conflict of interest, entrusting an independent researcher with the main coordination of the Delphi study is advisable. | 5 |
| *Interpretation and processing of results* | 6 | Consensus does not necessarily imply the ‘correct’ answer or judgement; (non)consensus and stable disagreement provide informative insights and highlight differences in perspectives concerning the topic in question. | 5 |
| *External validation* | 7 | It is recommended to have the final draft of the resulting guidance on best practice reviewed and approved by an external board or authority before publication and dissemination. | 6 |
| **Reporting** | | | |
| *Purpose and rationale* | 8 | The purpose of the study should be clearly defined and demonstrate appropriateness of use of the Delphi technique as a method to achieve the research aim. A rationale for the choice of the Delphi technique as the most suitable method needs to be provided. | 4 |
| *Expert panel* | 9 | Criteria for the selection of experts and transparent information on recruitment of the panel, sociodemographic details including information on expertise regarding topic in question and response rates over the ongoing iterations should be reported. | 5 |
| *Description of the methods* | 10 | The methods employed need to be comprehensible; this includes information on preparatory steps (How was available evidence on the topic in question synthesised?), piloting of material and survey instruments, design of the survey instrument(s), the number and design of survey rounds, methods of data analysis, processing and synthesis of experts’ responses to inform the subsequent survey round and methodological decisions taken by the research team throughout the process. | 5 |
| *Procedure* | 11 | Flow chart to illustrate the stages of the Delphi process, including a preparatory phase, the actual ‘Delphi rounds’, interim steps of data processing and analysis, and concluding steps. | S. Figure 1 |
| *Attainment of consensus* | 12 | It must be comprehensible how consensus was achieved through the process, including strategies to deal with non-consensus. | 5 |
| *Results* | 13 | Reporting of results for each round separately is highly advisable in order to make the evolving of consensus over the rounds transparent. This includes figures showing the average group response, changes between rounds, as well as any modifications of the survey instrument such as deletion, addition or modification of survey items based on previous rounds. | 5 |
| *Discussion of limitations* | 14 | Reporting should include a critical reflection of potential limitations and their impact of the resulting guidance. | 9 |
| *Adequacy of conclusions* | 15 | The conclusions should adequately reflect the outcomes of the Delphi study with a view to the scope  and applicability of the resulting practice guidance. | 10 |
| *Publication and dissemination* | 16 | The resulting guidance on good practice should be clearly identifiable from the publication, including recommendations for transfer into practice and implementation. If the publication does not allow for a detailed presentation of either the resulting practice guidance or the methodological features of the applied Delphi technique, or both, reference to a more detailed presentation elsewhere should be made (e.g. availability of the full guideline from the authors or online; publication of a separate paper reporting on methodological details and particularities of the process (e.g. persistent disagreement and controversy on certain issues)). A dissemination plan should include endorsement of the guidance by professional associations and health care authorities to facilitate implementation. | 11 |

**Supplemental Table 2:** Phase III serial focus group and partnership meeting objectives

| **Meeting Type & Number** | **Participant Type & Number** | **Objective** |
| --- | --- | --- |
| *Focus Group 1* | Youth (n=4) and parents (n=4) | To review, revise, and refine the consensus statements. |
| *Focus Group 2* | Researchers (n=5) | To review, revise, and refine the consensus statements. |
| *Focus Group 3* | Healthcare providers (n=3) | To review, revise, and refine the consensus statements. |
| *Partnership Meeting 1* | Youth (n=3) and experts (n=2) | To interpret feedback on the consensus statements. |
| *Focus Group 4* | Youth (n=4) and parents (n=4) | To provide feedback on the layout, colour scheme, font choices, and general visual appeal of the digital tool prototype. |
| *Focus Group 5* | Researchers (n=5) | To ensure the information provided in the digital tool prototype was accurate, valid, and age and grade-level appropriate. |
| *Focus Group 6* | Healthcare providers (n=3) | The ensure the exercises provided in the digital tool prototype were accessible, appropriate, and evidence based. |
| *Partnership Meeting 2* | Youth (n=3) and experts (n=2) | To interpret feedback on the digital tool prototype. |
| *Focus Group 7* | Youth (n=4) and parents (n=4) | To provide feedback on the final digital tool. |
| *Partnership Meeting 3* | Youth (n=3) and experts (n=2) | To interpret feedback on the final digital tool. |

**Supplemental Table 3.** Phase III modified Delphi consensus results by round, domain, and theme

| **ADULTS** | | | | | | | | | |
| --- | --- | --- | --- | --- | --- | --- | --- | --- | --- |
| **Domain & Themes** | **Round 1** | | | | **Round 2** | | | | |
|  | *Initial items* | *Consensus N* | *Consensus %* | *New Items* | *Initial Items* | *Consensus N* | *Consensus %* | *Excluded N* | *Excluded %* |
| **Domain 1** | 21 | 7 | 33.33% | 7 | 20 | 9 | 45.00% | 11 | 55.00% |
| Theme 1 | 11 | 2 | 18.18% | 5 | 14 | 6 | 42.86% | 8 | 57.14% |
| Theme 2 | 10 | 5 | 50.00% | 2 | 6 | 3 | 50.00% | 3 | 50.00% |
| **Domain 2** | 23 | 18 | 78.26% | 6 | 11 | 6 | 54.55% | 5 | 45.45% |
| Theme 3 | 13 | 10 | 76.92% | 4 | 7 | 3 | 42.86% | 4 | 57.14% |
| Theme 4 | 5 | 4 | 80.00% | 1 | 2 | 1 | 50.00% | 1 | 50.00% |
| Theme 5 | 5 | 4 | 80.00% | 1 | 2 | 2 | 100.00% | 0 | 0.00% |
| **Domain 3** | 24 | 4 | 16.67% | 10 | 30 | 27 | 90.00% | 3 | 10.00% |
| Theme 6 | 6 | 3 | 50.00% | 1 | 4 | 4 | 100.00% | 0 | 0.00% |
| Theme 7 | 14 | 0 | 0.00% | 7 | 21 | 20 | 95.24% | 1 | 4.76% |
| Theme 8 | 4 | 1 | 25.00% | 2 | 5 | 3 | 60.00% | 2 | 40.00% |

| **YOUTH** | | | | | | | | | |
| --- | --- | --- | --- | --- | --- | --- | --- | --- | --- |
| **Domain & Themes** | **Round 1** | | | | **Round 2** | | | | |
|  | *Initial items* | *Consensus N* | *Consensus %* | *New Items* | *Initial Items* | *Consensus N* | *Consensus %* | *Excluded N* | *Excluded %* |
| **Domain 1** | 21 | 13 | 61.90% | 13 | 21 | 16 | 76.19% | 5 | 23.81% |
| Theme 1 | 11 | 6 | 54.55% | 9 | 14 | 10 | 71.43% | 4 | 28.57% |
| Theme 2 | 10 | 7 | 70.00% | 4 | 7 | 6 | 85.71% | 1 | 14.29% |
| **Domain 2** | 23 | 19 | 82.61% | 7 | 11 | 9 | 81.82% | 2 | 18.18% |
| Theme 3 | 13 | 11 | 84.62% | 2 | 4 | 2 | 50.00% | 2 | 50.00% |
| Theme 4 | 5 | 3 | 60.00% | 2 | 4 | 4 | 100.00% | 0 | 0.00% |
| Theme 5 | 5 | 5 | 100.00% | 3 | 3 | 3 | 100.00% | 0 | 0.00% |
| **Domain 3** | 24 | 17 | 70.83% | 6 | 13 | 12 | 92.31% | 1 | 7.69% |
| Theme 6 | 6 | 5 | 83.33% | 4 | 5 | 4 | 80.00% | 1 | 20.00% |
| Theme 7 | 14 | 10 | 71.43% | 2 | 6 | 6 | 100.00% | 0 | 0.00% |
| Theme 8 | 4 | 2 | 50.00% | 0 | 2 | 2 | 100.00% | 0 | 0.00% |

**Supplemental Figure 1:** Modified Delphi consensus process flow diagram

68 **items** to be rated among youth and adults

0 **items** rated not essential

29 **items** rated essential

by adult consensus

0 **items** rated not essential

49 **items** rated essential

by youth consensus

**ROUND 1**

22 new **items** suggested from adults

26 new **items** suggested from youth

45 **items** to be rated among youth

61 **items** to be rated among adults

8 **items** did not reach consensus among youth

19 **items** did not reach consensus among adults

**ROUND 2**

86 final **consensus items** from youth

71 final **consensus items** from adults

**PHASE III**

21 final **consensus statements**

among youth and adults

*Understanding mental health, 4*

*Exercising mental health, 6*

*Engaging with mental health support, 8*

*Evaluating digital mental health support, 3*
